# GSDMD-Dependent Neutrophil Extracellular Traps Mediate Portal Vein Thrombosis and Associated Fibrosis in Cirrhosis

**DOI:** 10.3390/ijms25169099

**Published:** 2024-08-22

**Authors:** Ying Che, Youjung Chien, Yuli Zhu, Xiaoquan Huang, Ling Wu, Yingjie Ai, Siyu Jiang, Feng Li, Shiyao Chen

**Affiliations:** 1 Department of Gastroenterology and Hepatology, Zhongshan Hospital, Fudan University, Shanghai 200032, China; 21111210014@m.fudan.edu.cn (Y.C.); 22111210182@m.fudan.edu.cn (Y.C.);; 2 Department of Ultrasound, Zhongshan Hospital, Fudan University, Shanghai 200032, China; 3 Endoscopy Center and Endoscopy Research Institute, Zhongshan Hospital, Fudan University, Shanghai 200032, China; 4 Center of Evidence-Based Medicine, Fudan University, Shanghai 200032, China

**Keywords:** portal vein thrombosis, cirrhosis, neutrophil extracellular traps, immunothrombosis, myofibroblast

## Abstract

Portal vein thrombosis (PVT) is a challenging and controversial complication of cirrhosis. Experimental models that reproduce cirrhotic PVT and effective pharmacological therapies are limited. We aimed to investigate the nature course and mechanisms of PVT in cirrhosis. A novel PVT model was developed via two-step total portal vein ligation in healthy and thioacetamide (TAA)-cirrhotic rats. Circulating and liver-infiltrating neutrophils were isolated from individuals with cirrhosis to examine neutrophil extracellular traps (NETs) and explore their unique characteristics and implications in PVT-associated fibrosis in cirrhosis. We further validated macrophage–myofibroblast transition (MMT) via multiplex immunofluorescence and single-cell sequencing. In the experimental model, cirrhosis promoted PVT development and portal vein intimal thickening. Interestingly, cirrhosis promoted spontaneous resolution of PVT due to instability of thrombus structure, along with pulmonary and intrahepatic clots. NETs-MMT mediate cirrhotic PVT and PVT-associated fibrosis, including fibrotic thrombus remodeling and increased hepatic collagen deposition. Mechanistically, caspase-4-dependent activation of neutrophils and GSDMD mediated the formation of NETs. The extracellular DNA of NETs promoted TGF-β1/Smad3-driven MMT. Inhibiting GSDMD with disulfiram suppressed cirrhotic PVT and prevented associated fibrosis. The cirrhotic PVT model reflected the following three main characteristics of cirrhotic PVT: spontaneous resolution, immunothrombosis, and intimal fibrosis. Targeting NETs with GSDMD inhibitors may serve as a new therapeutic concept to treat cirrhotic PVT.

## 1. Introduction

Portal vein thrombosis (PVT) is unique among the subtypes of deep vein thrombosis (DVT) [1]. The portal vein drains gastrointestinal blood into the hepatic sinusoids but not the heart and lacks pulsatile flow that is influenced by the cardiac cycle [2]. It does not possess valves, which are the points of origin of DVT [3]. The low-pressure, slow-flow, and high-volume hemodynamics of the portal venous system represent a distinctive vascular environment, making thrombosis a rare event in the general population. Cirrhosis is the most common single cause of PVT, with its prevalence increasing as the disease severity, ranging from 10% to 26% [4]. The common site of thrombosis in cirrhosis is also the portal vein, rather than the deep vein and pulmonary circulation [5]. Recent research has found that cirrhotic PVT consists of thickening of the portal vein wall intima, with an additional fibrin-rich thrombus in only one-third of cases [6]. These features indicate that cirrhotic PVT is a challenging and uniquely distinctive entity.

The extent to which PVT influences the outcomes of cirrhosis has been extensively debated. Some studies have shown that PVT can aggravate portal hypertension in patients with cirrhosis, which results in an increased risk of gastrointestinal bleeding and decompensation [7,8]. Other studies demonstrated that the development of PVT does not predict liver disease progression or outcome [9]. In contrast, it is associated with liver transplant and post-transplant significant mortality [10]. This controversial issue is further complicated by approximately 40% of cirrhotic patients experiencing spontaneous resolution of PVT even without treatment, a phenomenon rarely observed in non-cirrhotic populations [4,11,12]. Hence, there is a pressing need to comprehend the mechanisms of cirrhotic PVT and elucidate its impact on the portal vein region and the liver.

The distinctive and intricate feature of the portal vein system presents significant challenges for developing an animal model of PVT. Despite several experimental PVT models being reported, none of them simulate the whole pathological process of this disease [13,14,15,16]. The pathogenesis of thrombosis is multifactorial and is explained by Virchow’s triad: venous stasis, hypercoagulability, and vessel injury [17]. The available data established decreased portal flow velocity as one of a few clear predictive factors in cirrhotic PVT, but the data do not support the role of hypercoagulability [18,19]. Thus, we sought to achieve portal vein stasis via total portal vein ligation, mimicking the clinical scenario of decreased portal flow velocity. Therefore, we developed a cirrhotic PVT model in TAA-cirrhotic rats by total portal vein ligation.

Current ultrasound elastography techniques typically assume tissues are homogeneous and purely elastic, employing simplistic linear models to evaluate tissue elasticity. However, liver and thrombus exhibit viscoelastic properties, combining characteristics of both elastic solids and viscous fluids [20]. In addition, the viscoelastic properties of thrombus affect the occlusion or rupture of blood clots [21]. Commercially available Shear Wave Elastography (SWE) not only quantifies tissue elasticity but also provides Shear Wave Dispersion (SWD) to assess tissue viscosity in its updated version, in which viscosity is closely linked to necrosis and inflammation [22]. Hence, we quantified the viscoelasticity of the liver and thrombus to evaluate their structure and inflammation.

Immunothrombosis is a type of host defense mechanism, implicated in trapping pathogens to restrict their systemic spreading. However, in some cases, this mechanism is extensively activated, leading to thrombotic events [23]. As a response to inflammatory stimuli, neutrophils produce neutrophil extracellular traps (NETs), which are extracellular fibers primarily composed of DNA and histones that carry myeloperoxidase (MPO) and neutrophil elastase [24]. Although intact neutrophils may not be present in chronic inflammatory lesions, the NETs released by them may amplify the inflammatory response beyond their short life-span in tissues. NETs not only affect thrombus growth and stability by serving as a scaffold but can also act as an activating stimulus for thrombus generation [25,26,27,28]. During thrombus resolution, macrophages are recruited to secrete enzymes, cytokines, and growth factors that facilitate clot degradation and tissue remodeling. Notably, macrophages display high plasticity and functional diversity [29]. Myofibroblasts are the primary cell type responsible for the synthesis and deposition of fibrillar collagen deposition during tissue fibrosis. acrophage-to-myofibroblast transition (MMT) is characterized by macrophages directly differentiating into myofibroblasts and secreting extracellular matrix proteins, including type I collagen. It can promote fibrosis in multiple organ systems, such as the lungs [30], kidneys [31], heart [32], and atherosclerotic plaque [33]. 

In this study, we developed a novel rat model of PVT to produce thrombi consistent with the structural characteristics of human PVT. This model effectively mimics the progression of PVT from acute to chronic inflammation and the subsequent vein wall remodeling. Our findings identify a previously unrecognized role of NETs-MMT in cirrhotic PVT and associated fibrosis, as well as novel insights into the development of novel therapies.

## 2. Results

### 2.1. Natural Course of PVT in the New Model

**Figure 1:**
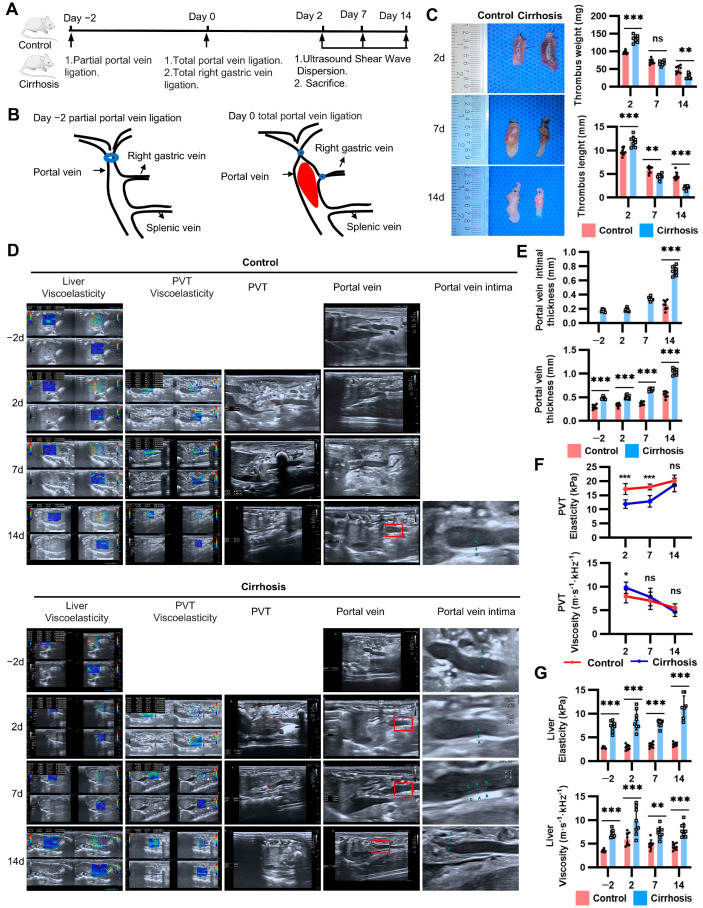
Natural course of PVT in the new model. (**A**) Experimental flowchart demonstrating two-stage total portal vein ligation performed by days −2 and 0, and assessment of PVT and liver using Ultrasound Shear Wave Dispersion. (**B**) Schematic illustration: First step: 70% of the portal vein was ligated by day −2. Second step: total portal vein and right gastric vein ligation were performed to produce an occlusive thrombus by day 0. (**C**) Left, representative images of PVT harvested from control and cirrhosis groups by days 2, 7, and 14. Right, measurements of the thrombus weight and length. (**D**) Representative images and (**E**–**G**) scattered bar graph quantifications: In vivo assessment of the viscoelasticity of the liver and PVT, portal vein wall in control and cirrhosis groups via ultrasound by days −2, 2, 7, and 14. The intimal thickness was too thin to measure in the control group by days −2, 2, and 7. The SWD and SWE indicate elasticity and viscosity, respectively. Red dotted lines demarcate the thrombus. The red boxes indicate the portal vein and its intima. In (**C**,**E**,**G**), each dot represents a single rat. Data represent mean with SEM. Statistical significance was determined by two-way ANOVA. * *p* < 0.05, ** *p* < 0.01, *** *p* < 0.001.

To investigate the natural history and underlying mechanisms of PVT, we developed a new rat model that reproduced the process of thrombosis and its resolution. To ensure the formation of collateral vessels and prevent fulminant hepatic failure, partial ligation of the portal vein resulting in 70% stenosis was initially performed. Two days later, simultaneous ligation of the portal vein and left gastric vein was performed to achieve complete portal vein stasis (Figure 1A,B). Notably, this model achieved almost 100% incidence of PVT in both groups. The mortality rates were 2.5% (1/40) and 6% (3/50) in the control and cirrhosis groups, respectively, with the main cause being anesthesia accident. Compared with the control rats, the cirrhotic rats exhibited higher thrombus weights and lengths with accelerated resolution (Figure 1C). Then, we further quantified the thickness of the portal vein wall and the viscoelasticity of the liver and thrombus (Figure 1D). At baseline, the thickness of the portal vein intima and wall in the cirrhotic rats was greater than that in the control rats. The portal vein wall and intima were further thickened during thrombus resolution (Figure 1E). The elasticity of the thrombus in the cirrhosis group decreased, while the viscosity increased compared with the control group by day 2. In addition, the elasticity increased and viscosity diminished as a function of thrombus age (Figure 1F). In the context of PVT, the elasticity and viscosity of the liver in cirrhotic rats were significantly higher than those in control rats (Figure 1G). Altogether, these results demonstrated that cirrhosis promoted the formation of “softer” PVT and supported the spontaneous resolution of PVT. The combination of cirrhosis and PVT contributed to the thickening of the portal vein wall.

### 2.2. Thrombus Instability Explained the Spontaneous Resolution of PVT in Cirrhosis

**Figure 2:**
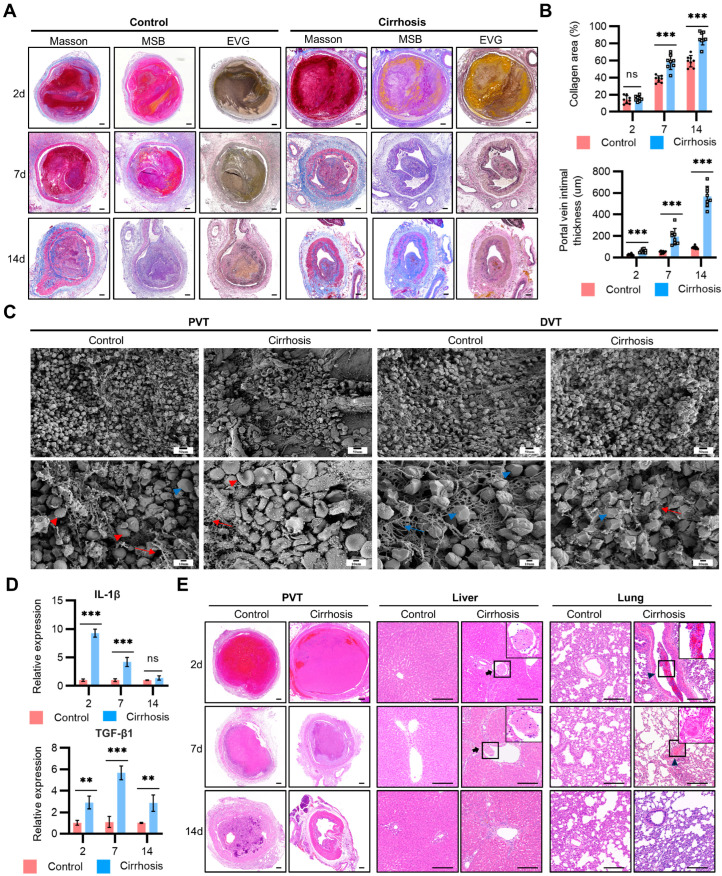
Thrombus instability explained the spontaneous resolution of PVT in cirrhosis. (**A**) Representative images of PVT stained for collagen with Masson’s Trichrome (Masson), Martius Scarlet Blue (MSB), and Elastic Van Gieson (EVG) for elastin in both groups by days 2, 7, and 14. Scale bar = 200 µm (**B**) Quantification of portal vein intimal thickness and average percent thrombus occupied by collagen in both groups by days 2, 7, and 14. The collagen area was dyed blue by Masson, and the intimal thickness was determined with EVG. Each dot represents a single rat. (**C**) Effect of cirrhosis and the portal vein on the thrombus structure. Representative SEM images of PVT and DVT samples from the control and cirrhotic rats by day 2 post-thrombosis. Biconcave-shaped red blood cells (red arrowhead), polyhedral erythrocytes (blue arrowhead), fibrin networks (red arrow), and fibrin bundle (blue arrow). (**D**) QPCR analysis for genes IL-1β and TGF-β1 (*n* = 4–6 rats per group) of PVT in both groups by days 2, 7, and 14. (**E**) Representative H&E staining images of PVT, lung, and liver of rats in the control and cirrhosis groups by days 2, 7, and 14 to distinguish the correlation among hepatic thrombi, pulmonary embolism, and PVT in cirrhosis. In the clots, the dark-red-stained area is RBC-rich, whereas the area of fibrin/platelets is stained light pink. Arrow indicates intrahepatic thrombosis. Triangle indicates pulmonary thrombosis. Scale bar = 200 µm. In (**B**,**D**), data represent the mean with SEM. Statistical significance was determined by two-way ANOVA. ** *p* < 0.01, *** *p* < 0.001.

To distinguish between poor endothelial adherence and structural instability as factors in the spontaneous resolution of cirrhotic PVT, we further evaluated the histopathology and composition of the thrombi. Histological staining revealed reduced fibrin and increased collagen in cirrhotic PVT compared with non-cirrhotic PVT over a period of 14 days. Moreover, the fibrotic intima of the portal vein in cirrhosis initiated as early as day 2 following thrombus formation, and persistently increased, consistent with the process of thrombus fibrosis. It is clear that the resolving thrombus incorporated into the portal vein wall as it healed, which created a thickened intimal (Figure 2A,B). Then, we analyzed the influence of cirrhosis and the portal vein (compared with vena cava) on thrombus structure by scanning electron microscopy. Compared with the control rats, the cirrhotic rats displayed increased fibrin density and decreased fibrin fiber thickness in the thrombi (PVT and DVT). Aligning with a previous clinical study [6], polyhedral erythrocytes (a marker for clot contraction) rarely exist in PVT in contrast to DVT (Figure 2C). These data suggest that cirrhosis and the portal vein impede the structural stability of thrombi. Simultaneous upregulation of the genes associated with inflammation (IL-1β) and fibrosis (TGF-β1) was also detected (Figure 2D). Considering the risk of thrombus dislodgement and embolism, we found apparent pulmonary and intrahepatic clots in the cirrhotic rats with PVT by days 2 and 7. The pulmonary clots had a similar histological structure to PVT, characterized by alternating layers of fibrin and erythrocytes, with a notable prevalence of erythrocytes. Different from PVT, intrahepatic clots were rich in fibrin and deficient in red blood cells (Figure 2E). We speculated that the pulmonary clots were disappeared thrombi that embolized to the lung ectopic embolism, while the intrahepatic clots arose locally through de novo thrombosis in a hypercoagulable state in the animal model. Consequently, these data suggested that thrombus instability accounts for the spontaneous resolution of cirrhotic PVT, which may be at the expense of ectopic embolism.

### 2.3. NETs Mediate PVT and Associated Inflammation in Cirrhosis

**Figure 3:**
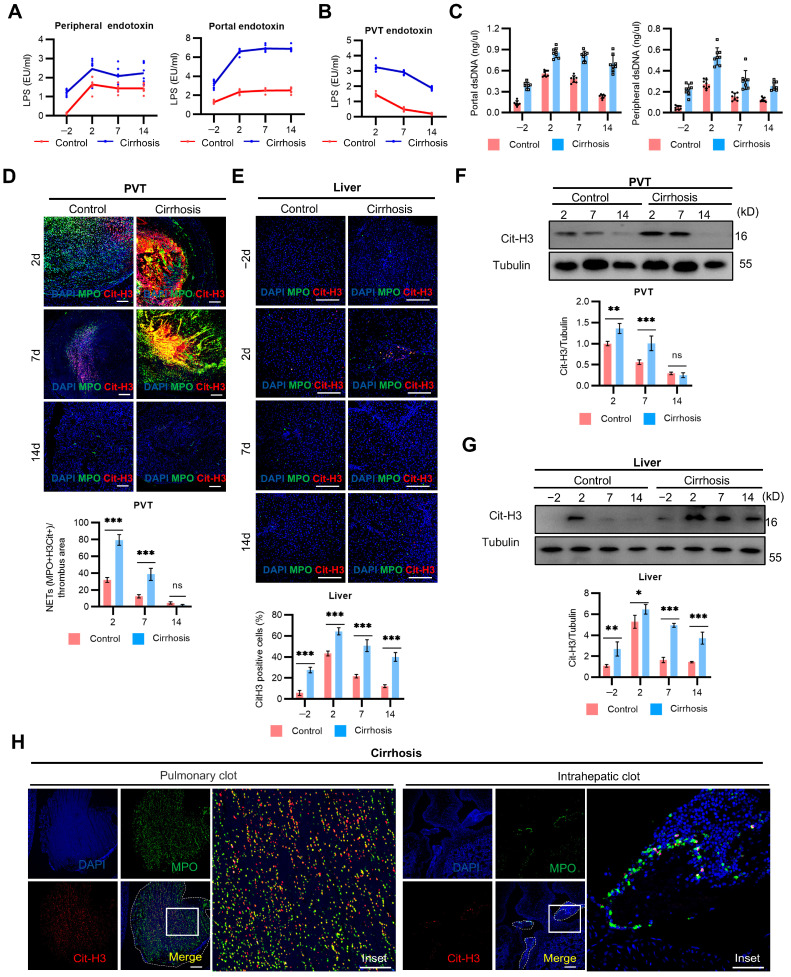
NETs mediate PVT and associated inflammation in cirrhosis. (**A**) LPS levels of portal plasma, systemic plasma, and PVT (**B**) in both groups by days −2, 2, 7, and 14. (**C**) Extracellular dsDNA in the portal and peripheral plasma in both groups by days −2, 2, 7, and 14 (n = 8 rats per group). Each dot represents a single rat. (**D**) Representative immunofluorescence staining for typical NET markers (Cit-H3, MPO) in the PVT from both groups by days 2, 7, and 14. Scale bar = 200 µm (**E**) Representative immunofluorescence staining for NETs in the liver from both groups by days −2, 2, 7, and 14. Scale bar = 200 µm. (**F**) Western blotting analysis of typical NET marker (Cit-H3) protein levels in the PVT from both groups by days 2, 7, and 14. (**H**) Representative immunofluorescence staining for a typical NET marker in pulmonary and intrahepatic clots from the cirrhotic groups by day 2. In (**D**,**E**), bar graph quantifications are shown right below the respective histochemistry panels. In (**F**,**G**), bar graph quantifications are shown right below the respective Western blot. Statistical significance was determined by two-way ANOVA. * *p* < 0.05, ** *p* < 0.01, *** *p* < 0.001.

Recent research has indicated that local alterations of the portal vein in cirrhosis, including bacterial translocation and endotoxemia, may be responsible for the increased risk of thrombosis in the portal system than in systemic venous circulation [34]. The LPS level in the portal and peripheral serum of the cirrhotic rats was significantly higher than that in those of the control rats (Figure 3A). Similarly, cirrhotic PVT captured more LPS than non-cirrhotic PVT (Figure 3B). Extracellular dsDNA showed a significant increase in the portal and peripheral vein of the cirrhotic rats with PVT (Figure 3C). Then, the expression of NETs in the PVT and liver of the cirrhotic rats was significantly higher than that in the control rats (Figure 3D,E). There were persistent NETs infiltration in the cirrhotic rats with PVT, which is not parallel to the progress of thrombolysis. These findings were further confirmed by the expression of H3-cit (Figure 3F,G). Furthermore, the NETs present in the pulmonary clots exhibited structural similarities to those observed in PVT, while intrahepatic clots were characterized by punctate NETs (Figure 3H). The above results indicated that portal LPS facilitates the persistent infiltration of NETs formation in thrombi and the liver in cirrhosis. 

### 2.4. Caspase-4-Dependent Activation of Neutrophil GSDMD Promotes NETs Formation in Cirrhosis

**Figure 4:**
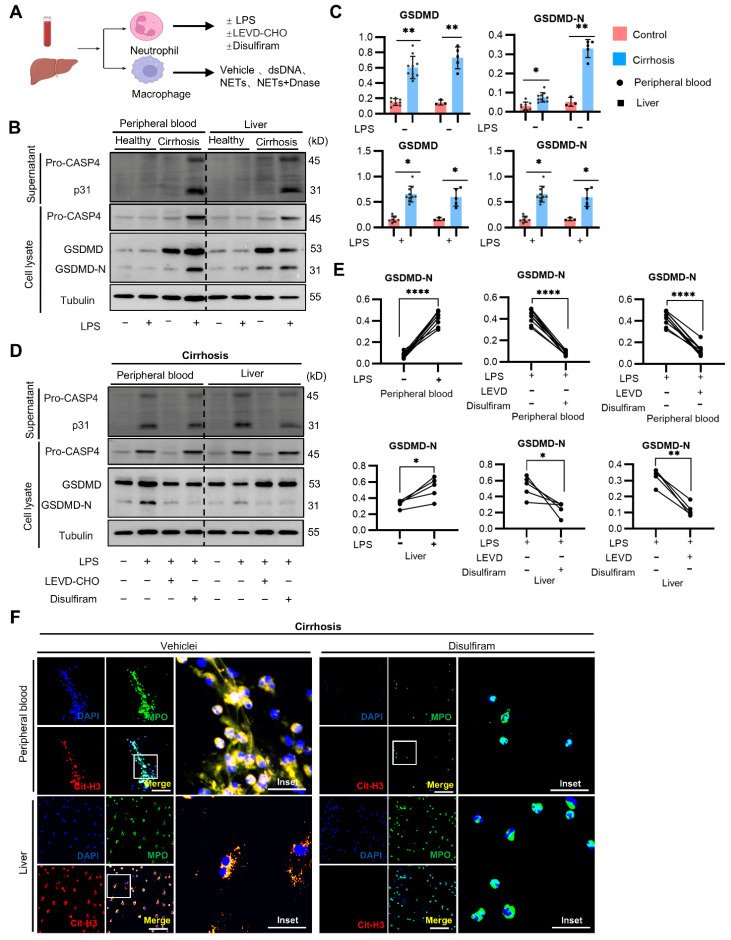
Caspase-4-dependent activation of neutrophil-GSDMD promotes NETs formation in cirrhosis. (**A**) Experimental scheme: control and cirrhotic human liver tissues and blood were subjected to neutrophil and macrophage isolation. Neutrophils were treated with LEVD-CHO (caspase-4 inhibitor, 20 μM), disulfiram (GSDMD inhibitor, 30 µM), or vehicle 1 h before stimulation with LPS (10 µg/mL) for 4 h. (**B**) Representative Western blot micrograph showing uncleaved GSDMD and cleaved GSDMD-N, caspase-4, processed caspase-4, and Cit-H3 in the neutrophils of healthy individuals and cirrhotic patients with or without LPS. (**C**) Densitometric analyses of Western blot micrographs (representative example shown in panel b). (**D**) Representative Western blot micrograph showing uncleaved GSDMD, cleaved GSDMD-NT, caspase-4, processed caspase-4, and Cit-H3 in the neutrophils from cirrhotic patients with or without LEVD-CHO or disulfiram or vehicle. (**E**) Densitometric analyses of Western blot micrographs (representative example shown in panel (**D**)). (**F**) NETs production through immunofluorescence staining in circulating and liver-infiltrating neutrophils of cirrhotic patients with or without disulfiram. Scale bars = 100 µm. For (**C**,**E**), each dot represents a separate human subject. Mean ± SEM shown in (**C**) are compared using Student *t* test. Data in (**E**) are compared using paired Student t test. * *p* < 0.05, ** *p* < 0.01, **** *p* < 0.0001.

We aimed to explore the mechanisms underlying NETs generation in individuals with cirrhosis (Figure 4A). The protein expression of uncleaved GSDMD in neutrophils isolated from the individuals with cirrhosis was significantly higher than that in the healthy controls with or without LPS incubation. Meanwhile, a higher expression of the GSDMD-N terminal fragment (GSDMD-N) was observed in the liver-infiltrating neutrophils of the cirrhosis group. Incubation with LPS further significantly increased the GSDMD-N expression of neutrophils in the cirrhotic individuals. These findings confirmed a chronic state of activation of liver-infiltrating neutrophils in cirrhosis. Recently, LPS has been shown to promote caspase-4-dependent cleavage of GSDMD in human macrophages [35]. We found that the expression of full-length and processed caspase-4 was also significantly higher in neutrophils from the individuals with cirrhosis with LPS incubation (Figure 4B,C). This result also suggested that caspase-4 auto-proteolysis is crucial for GSDMD cleavage. Finally, GSDMD-N was significantly reduced in neutrophils isolated from the cirrhotic individuals incubated with LPS and LEVD-CHO or disulfiram compared with LPS alone (Figure 4D,E). LPS triggered substantial NETs from circulating neutrophils, as observed by nuclear delobulation, DNA extrusion, DNA-MPO colocalization, and histone H3 citrullination. In contrast, liver-infiltrating neutrophils induced the formation of spot-like NETs with ruptured cell membranes and complete nuclear membranes. We further explored whether disulfiram can prevent NET release. The presence of disulfiram in both the liver and peripheral blood led to a significant reduction in NET formation (Figure 4F). These results suggested that caspase-4-dependent activation of neutrophil-GSDMD promotes NET production in cirrhosis, which can be inhibited by disulfiram.

### 2.5. NETs Directly Promote Macrophage to Myofibroblast Transdifferentiation in Cirrhosis

**Figure 5:**
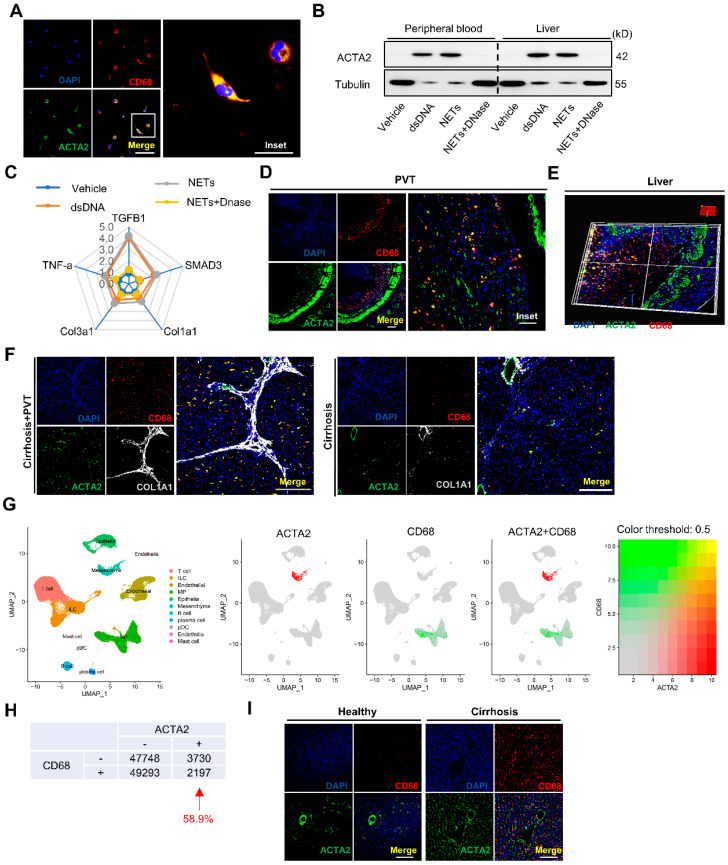
NETs directly promote macrophage-to-myofibroblast transdifferentiation in cirrhosis. (**A**) Representative immunofluorescent staining of ACTA2 (green) and CD68 (red) in monocytes exposed to NETs. Scale bars = 100 µm. (**B**) Western blot analysis for the ACTA2 expression of macrophages after coincubation with NETs, NETs plus DNase, or vehicle. (**C**) QPCR analysis for TGF-β signaling pathway genes in differentiated cells. Data are shown as mean ± SEM. (**D**) Representative immunofluorescence staining for MMT by co-expressing CD68 (red) and ACTA2 (green) in PVT from cirrhotic rats by day 7. Scale bar = 200 μm. (**E**) Reconstructed 3D modeling further demonstrates spindle-like myofibroblast morphology of MMT cells in the livers of cirrhotic rats with PVT by day 2. (**F**) Representative three-color immunofluorescence staining for CD68 (red), ACTA2 (green), and COL1A1 (white) in the livers of cirrhotic rats with PVT and without PVT. Scale bar = 200 µm. (**G**) TSNE plots showing cell populations (left panel) and expression of ACTA2 and CD68 (right panel) separately and superimposed together in the dataset. (**H**) Stratification of cells based on their levels of expression of ACTA2 and CD68. (**I**) Confocal imaging detected MMT in the liver from healthy individuals and cirrhotic patients. Scale bars = 200 µm.

Next, we investigated the effects of NETs and NET-associated components on MMT. To this end, macrophages isolated from patients with cirrhosis were co-incubated with either dsDNA, isolated NETs, or NETs plus DNase. In the presence of NETs, differentiated cells exhibited mixed morphology (round and spindle-shaped), with co-expression of ACTA2 and CD68 (Figure 5A). NETs promoted the expression of ACTA2 in macrophages (Figure 5B). QPCR analysis revealed significant upregulation of the TGF-β signaling pathway-associated genes (Figure 5C). Considering the dynamic plasticity of macrophage functions in cirrhosis, we next traced the phenotypic and functional changes in macrophages during and after infiltrating into the thrombus and liver. There was an abundance of MMT phenotype cells co-expressing myofibroblast marker ACTA2 and macrophage marker CD68 in PVT (Figure 5D) and liver (Figure 5E). The coimmunostaining for ACTA2, CD68, and Col1a1 revealed that macrophages directly contribute to the production of collagen in a time-dependent pattern. Notably, MMT was also found in the cirrhotic rats without PVT (Figure 5F). Thus, we examined publicly available single-cell RNA sequencing datasets (GSE136103) from patients with cirrhosis [36]. Analysis of these datasets showed that 58.9% of the ACTA2-positive cells (myofibroblasts) also expressed CD68 (Figure 5G,H). The presence of MMT was further confirmed in the cirrhotic individuals, whereas it was absent in the healthy volunteers (Figure 5I). Together, these results indicate that NET-dependent macrophage differentiation leads to a predominantly fibroblast phenotype, which contributes to thrombus remodeling and hepatic collagen deposition in cirrhosis.

### 2.6. GSDMD Inhibition with Disulfiram Inhibited Cirrhotic PVT and Associated Fibrosis

**Figure 6:**
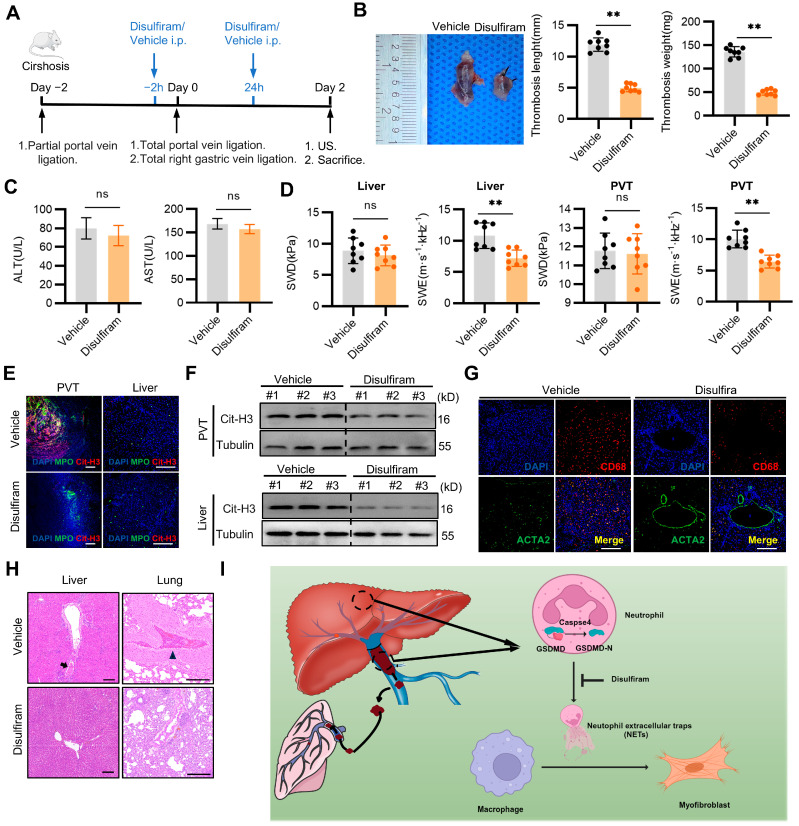
GSDMD inhibition with disulfiram inhibited cirrhotic PVT and associated fibrosis. (**A**) Experimental flowchart: Cirrhotic rats were administered two doses of the GSDMD inhibitor, disulfiram (80 mg/kg body weight each dose, intraperitoneally), the first dose 2 h before the second surgery, and the second dose 24 h after portal vein total ligation. Control mice were administered with a control vehicle. Forty-eight hours following portal vein total ligation, the rats were killed, and thrombus formation was evaluated. (**B**) Left, representative images of PVT in both groups by day 2. Right, measurements of the thrombus weight and length. (**C**) Plasma alanine aminotransferase (ALT) and aspartate aminotransferase (AST) from cirrhotic rats treated with disulfiram and vehicle by day 2. (**D**) The SWE and SWD of livers and PVT in both groups via ultrasound by day 2. (**E**) Immunofluorescence staining for NETs in PVT and livers from both groups by day 2. (**F**) Western blotting analysis of Cit-H3 protein levels in PVT and livers from both groups by day 2. (**G**) Representative immunofluorescent staining of ACTA2 (green) and CD68 (red) in livers from both groups. Scale bars = 200 µm. (**H**) Representative H&E staining images of the lungs and livers in both groups by day 2. In (**B**,**D**), each dot represents a single rat. Data represent mean with SEM. Statistical significance was determined by paired Student t test. ** *p* < 0.01. (**I**) Schematic diagram of this study: Cirrhosis promotes the formation and the spontaneous resolution of PVT, along with pulmonary and intrahepatic clots. Caspase-4/GSDMD-dependent neutrophil extracellular traps mediate how macrophages directly transdifferentiate into myofibroblasts, which leads to the production of collagen, and, furthermore, the aggravation of thrombus remodeling and hepatic collagen deposition. Inhibition of GSDMD mitigates cirrhotic PVT and prevents the associated fibrosis.

Next, we investigated whether the inhibition of GSDMD with disulfiram could serve as a potential therapeutic strategy for cirrhotic PVT. Briefly, the cirrhotic rats were administered disulfiram intraperitoneally, at a dose of 80 mg/kg, 2 h before and 24 h after the total portal vein ligation-induced PVT (Figure 6A). GSDMD inhibition decreased the initial thrombus lengths and weights by day 2 (Figure 6B). There was no significant difference in ALT and AST between disulfiram and the control group, and the side effects of drug hepatotoxicity were excluded (Figure 6C). The viscosity of the liver and thrombi were significantly lower in the disulfiram-treated rats compared with the vehicle-treated rats, which indicated disulfiram ameliorates infiltrating inflammation (Figure 6D). Disulfiram significantly attenuated NET formation in both PVT and the liver (Figure 6E,F). Furthermore, disulfiram effectively suppressed the process of MMT via inhibiting NET formation (Figure 6G). Intriguingly, treatment with disulfiram blocked the formation of pulmonary and intrahepatic clots (Figure 6H). Together, these data suggest that disulfiram could serve as a new therapeutic concept in cirrhotic PVT.

## 3. Discussion

The pathogenesis of PVT and its contribution to cirrhosis progression remain controversial. The novel rat model reflects the characteristics of spontaneous resolution, immunothrombosis, and intimal fibrosis of cirrhotic PVT. Interestingly, instability in the thrombus structure leads to the spontaneous resolution of cirrhotic PVT, accompanied by pulmonary and intrahepatic clots. Cirrhotic PVT further aggravates thrombus remodeling, forming intimal thickening and hepatic fibrosis via NETs-MMT. Mechanistically, caspase-4-dependent activation of neutrophil-GSDMD promotes the formation of NETs, which in turn mediated MMT. Furthermore, GSDMD inhibition with disulfiram inhibited cirrhotic PVT and prevented its sequelae. 

Compared with the existing PVT models [13,14,15], the rat model we developed here replicates the characteristics of cirrhotic PVT. The first step is the induction of the cirrhosis model by using chronic TAA injection [37]. The second step is total portal vein ligation, which mimics decreased portal vein blood flow in cirrhosis patients [18]. The model closely recapitulates the clinical features of cirrhotic PVT and possesses several advantages. First, the model provides a standardized approach for thrombus formation with low mortality rates. It demonstrates high reproducibility in thrombosis prevalence and thrombus size, without causing endothelial denudation or compromising vessel wall integrity. Second, this model accords with the natural history of PVT in patients with cirrhosis [4,11,12]. It reveals an intriguing paradox where cirrhosis fosters PVT development and expedites its spontaneous resolution. Third, the thrombus generated by this model is histologically similar to human PVT [6], characterized by a distinctive structure comprising biconcave erythrocytes entangled in a scaffold of relatively thin fibrin. Using this model, the comparison of PVT and DVT in cirrhotic rats and control rats further demonstrates that PVT is a special type of vein thrombus, and cirrhotic and non-cirrhotic PVT exhibited distinct organizational structures. Thus, we anticipate that this model is an adaptable tool that can be used to evaluate the risk factors, long-term progress, and treatment of cirrhotic PVT.

One of the major novel findings of the current study is that cirrhosis impedes the structural stability of PVT, thereby being accountable for the spontaneous resolution. Intimal fibrosis occurred even earlier than thrombotic fibrosis in the cirrhotic rats, reflecting firm adherence of the thrombus to the portal vein wall and ruling out poor endothelial adherence. Moreover, blood clot contraction is driven by fibrin–platelet meshwork and accompanied by compression of the biconcave erythrocytes to polyhedral erythrocytes [38]. The microstructure of cirrhotic PVT was characterized by finer fibrin and the absence of polyhedral erythrocytes, revealing impaired clot contraction. The impaired contraction potentially regulates the obstructiveness and embologenicity of a thrombus, resulting in larger and weaker PVT in cirrhosis. This poses a potential risk wherein the dissolved clot or its fragments may migrate to other places such as the lungs and liver. In the cirrhotic PVT model, the composition of a pulmonary clot is similar to PVT, whereas a hepatic thrombus differs. In fact, full ligation of the portal vein prevented the movement of thrombi to the intrahepatic vascular. However, portosystemic shunt and collateral circulation contributed to the dislodgement of thrombi into the pulmonary circulation. Therefore, in PVT patients with cirrhosis, a thrombus is more likely to be intravascularly embolized to the liver than the lung. Anticoagulation reduces all-cause mortality in patients with cirrhosis and PVT independently of recanalization, suggesting PVT may identify a group of patients with cirrhosis that benefit from long-term anticoagulation [39]. Combined with our research results, the survival benefit of anticoagulation may be related to the prevention of PVT-related macro- and microvascular thrombosis. Further clinical research is needed to explore whether the spontaneous resolution of PVT carries the risk of ectopic embolism, which may exceed the effects on portal obstruction.

The present results provided evidence for the active participation of PVT in immunity by its ability to capture and sequester LPS. An increase in portosystemic shunting leads to a higher concentration of LPS in the systemic circulation following the occurrence of PVT, thereby perpetuating the inflammatory response. Meanwhile, LPS continuously activates neutrophils to release NETs to amplify thrombotic inflammation. The formation of NETs by gut-derived LPS served as a trigger for cirrhotic PVT, and it is also involved in pulmonary and intrahepatic clots. Mechanistically, our current findings suggest that the sterile inflammatory milieu in cirrhosis promotes caspase-4-dependent activation of neutrophil-GSDMD, which leads to NETs generation in the thrombus and liver. Therefore, PVT is both driven by inflammation and generates inflammation.

Our data confirm that alterations in the portal vein wall are a direct result of cirrhosis and PVT. In the absence of PVT, animal experiments indicate that the portal vein wall is noticeably uneven and thickened in cirrhosis. Damage to the portal vein by altered shear stress related to portal hypertension and inflammation initiates vein wall fibrosis thickening, which may promote thrombus formation. Experimental models demonstrate that the pattern of PVT maturation incorporates the collagenous post-thrombotic tissue into the vein wall, forming a lesion similar to intimal hyperplasia. This process restores patency at the cost of portal vein fibrosis and non-compliant vein walls, resulting in uneven vessel thickening and potentially compromising vein functionality. The increased thickness of the portal vein wall can indicate compromised vein compliance and may be associated with portal hypertension to some extent. 

Cirrhosis is considered a multisystem disease, with immune dysfunction as the key to the progress of the disease. Macrophages proliferate locally within scar regions of liver tissue and stimulate hepatic stellate cells into myofibroblasts during chronic liver disease. In addition, portal vein fibroblasts are also one of the sources of myofibroblasts [40]. Here, we reported that NETs are able to transdifferentiate macrophages to myofibroblasts. It was unexpected that myofibroblasts derived from macrophages were significantly expressed in the liver of patients with cirrhosis, based on our reanalysis of public scRNA-seq data. In addition to producing extracellular matrix components, the contractile property of myofibroblasts also contributes to compressing thrombi and promoting their integration into the surrounding vein wall. Our work positioned MMT as a direct player that contributes to the fibrotic response following injury, promoting fibrosis associated with PVT, including increased hepatic collagen deposition and thrombus remodeling forming intimal thickening.

The only currently FDA-approved drug is the inhaled drug dornase alfa (recombinant DNase I) [41], which targets NETs that are already formed and released. However, DNase I has been reported to leave other crucial components of NETs, such as histones in situ or in the bloodstream, which can have detrimental effects [42]. A recent study showed that disulfiram, a classic drug used to treat alcoholism, is also a potent inhibitor of GSDMD at therapeutic doses. Disulfiram binds covalently to GSDMD, preventing pore formation in nuclear and plasma membranes to block the formation of new NETs [43]. Moreover, disulfiram has a well-established pharmacokinetic profile and a remarkable safety profile, as side effects have rarely been reported [44]. Building upon this evidence, we demonstrated that disulfiram-mediated inhibition of GSDMD in neutrophils effectively abolished NETs and blocked MMT in cirrhotic rats with PVT. Targeting events driven by NETs, rather than solely focusing on the coagulation cascade, provides a safe strategy for PVT while minimizing bleeding risks. Disulfiram’s ability to reduce the initial size of a thrombus and minimize the subsequent fibrotic response may lead to improved patient outcomes and a decreased risk of complications.

Our study has several limitations and requires further exploration in future research. First, although portal vein stasis generated thrombi with histological features resembling human PVT, it failed to fully recapitulate aspects of PVT pathogenesis in humans. Prospective clinical studies are needed to explore the best management and treatment of cirrhotic PVT. Second, although the effect of GSDMD inhibition by disulfiram may be associated with a reduction in NETs, we did not exclude its effect in other cells, such as macrophages.

In summary, our data provide details on distinctive phenotypes of PVT and shed light on the inflammation and fibrosis associated with PVT in cirrhosis. We found that macrophages acquire a fibroblast-like phenotype and directly contribute to collagen deposition in cirrhosis. Additionally, our findings showed that disulfiram could represent a new strategy for the treatment of PVT and associated fibrosis in patients with cirrhosis.

## 4. Materials and Methods

### 4.1. Animal Models of Cirrhosis

All animal study protocols were approved by the Institutional Animal Care and Use Committee (IACUC, 2023-025) of Zhongshan Hospital, Fudan University. Rats were maintained in a temperature-controlled environment (20–22 °C) with a 12 h light–dark cycle and free access to food and drinking water. Male Sprague–Dawley rats (150 g, Charles River, Shanghai, China) were intraperitoneally injected with 250 mg/kg body weight of thioacetamide (TAA, Sigma, St. Louis, MO, USA) two times a week for 12 weeks. Healthy male Sprague–Dawley rats (350 g, Charles River, Shanghai, China) were used for subsequent modeling and sample harvesting. At the end of the experimental period, liver sections of three rats in each group were randomly selected and stained with hematoxylin and eosin (H&E) and Sirius Red (SR) to confirm liver cirrhosis induced by TAA (Supplemental Figure S1).

### 4.2. PVT Model

Following the last TAA administration, the animals with cirrhosis underwent a detoxification period of 2–5 days before beginning portal vein ligation. The rats were anesthetized and maintained in 2–3% isoflurane (HEBEI YIPIN Pharmaceutical, China) and 2 L/min 100% oxygen during the procedure. Intra-operative and post-operative analgesia was achieved with a subcutaneous injection of buprenorphine (0.05 mg/kg/8 h for 48 h). After shaving and disinfection, a median laparotomy was performed, and the upper abdominal area was surgically prepared. First, partial portal vein ligation was performed as previously described [37]. In brief, a 20-gauge blunt-tipped needle was placed along the portal vein and ligated with a 4-0 silk suture all together. Subsequent removal of the needle resulted in a calibrated stenosis of the portal vein. After a 48 h period, the portal vein was completely ligated at the same site, simultaneously with the ligation of the left gastric vein.

A total of ninety rats were included in the present study. The mortality rates in the control and cirrhosis groups were 2.5% (1/40) and 6% (3/50), respectively, with the primary cause being anesthesia accidents. Among the forty control rats, the natural time course of PVT resolution was examined through harvests conducted at 2 days (n = 8), 7 days (n = 8), 14 days (n = 8), 21 days (n = 8), and 28 days (n = 7/8, one died in the perioperative period) after the procedure. Similarly, twenty-eight cirrhotic rats underwent surgery to investigate the natural time course of PVT resolution in the presence of cirrhosis, with harvests conducted at 2 days (n = 8), 7 days (n = 8), 14 days (n = 8), and 21 days (n = 5/8, three died in the perioperative period). Additionally, sixteen cirrhotic rats were operated on to evaluate the role of disulfiram. Thrombi were blotted with delicate task wipers to absorb and remove excess fluid and then weighed in milligrams using an analytical balance.

### 4.3. DVT Model

The rat model of DVT was induced by inferior vena cava (IVC) stasis. In brief, rats were anesthetized, and median laparotomy was performed. IVC was gently separated from the aorta and completely ligated with a silk suture immediately below the left renal vein. All visible side branches between the left renal vein and iliolumbar veins were completely ligated. The rats were sacrificed on day 2, and the abdominal IVC (infrarenal to the iliac bifurcation) was harvested. The thrombus was carefully removed from the IVC and rinsed in saline for further analysis.

### 4.4. Human Sample Collection

The human sample collection was conducted between October 2022 and May 2023 at Zhongshan Hospital with Research Ethics Committee approval (no. B2020-125). Written informed consent was obtained from all participants. The inclusion criteria were as follows: (i) patients diagnosed with cirrhosis; (ii) aged 18 years or older; and (iii) received a computed tomography (CT) scan to detect PVT. The exclusion criteria were as follows: (i) cavernous transformation of the portal vein; (ii) schistosomal cirrhosis (which is characterized by periportal fibrosis [45]); (iii) malignant tumor; and (iv) a history of liver transplantation or transjugular intrahepatic portosystemic shunt. Non-tumorous liver tissue from hepatic resections was used as a healthy control. In detail, liver tissues were collected from five patients with cirrhosis and three healthy patients without chronic liver disease. Venous blood was collected from ten patients with cirrhosis and eight healthy individuals without chronic liver disease.

### 4.5. Statistical Analysis

Graphs were drawn using GraphPad Prism 8.0. Statistical significance evaluation was calculated using Student’s t test and one-way ANOVA. N represents the number of samples in each group. All values were expressed as the mean ± SEM. *p* < 0.05 was considered indicative of statistical significance.

## Data Availability

All data are available from the corresponding author on reasonable request.

## Supplementary Materials

The following supporting information can be downloaded at https://www.mdpi.com/article/10.3390/ijms25169099/s1.
