# Supplementary material for: GSDMD-Dependent Neutrophil Extracellular Traps Mediate Portal Vein Thrombosis and Associated Fibrosis in Cirrhosis
Source: Int J Mol Sci. 2024 Aug 22;25(16):9099. doi: 10.3390/ijms25169099 (PMC11354441; doi:10.3390/ijms25169099)

GSDMD-Dependent Neutrophil Extracellular Traps Mediate Portal Vein Thrombosis and Associated Fibrosis in Cirrhosis

Supplementary materials

Materials and methods

Quantification of extracellular double-stranded DNA

DsDNA in plasma was quantified with a Qubit™ dsDNA Assay kit (Q32851, Invitrogen) following the manufacturer's instructions. The absorbance was measured with a microplate reader (Qubit™ Fluorometer, Thermo Fisher Scientific, Waltham, MA, USA).

Neutrophil isolation

Whole blood was layered over Polymorphprep™ and centrifuged for 20 min at 800 g. The granulocyte-rich layer and the peripheral blood mononuclear cell (PBMC) layer were separately collected. Neutrophils were isolated from the previously collected granulocyte-rich layer using a Human Neutrophil Enrichment Kit (StemCell) according to the manufacturer’s specifications. Neutrophil purity was assessed using an XN-350 Hematology Analyzer (Sysmex Corp, Kobe, Japan) and was generally above 95%.

NETs isolation

Neutrophils (5x106 cells/mL) were seeded into 6-well cell culture plates and stimulated with 25 ng/ml GM-CSF and 10 μg/ml LPS for 4 h at 37°C. The supernatant was discarded, and the wells were washed with PBS and treated with 10 U/μl of the restriction enzyme AluI (Roche) for 30 min at 37°C. The supernatant was collected and centrifuged at 300 g for 5 min at 4°C to remove whole cells and debris. The NETs-rich supernatant was collected and stored at -80°C. The DNA concentration in NETs was

measured using PicoGreen (Thermo Fisher Scientific) as described.

Macrophage isolation and stimulation

According to the manufacturer's protocol, monocytes were isolated from the previously collected PBMC layer by negative selection using a human monocyte enrichment kit (StemCell). Isolated monocytes were re-suspended in RPMI medium supplemented with 3% FBS, 100 µg/ml penicillin, 100 µg/ml streptomycin, 2.5 µg/ml Amphotericin B, 2 mM glutamine, and 25 ng/ml GM-CSF. For stimulation experiments, monocytes (1x106 cells/ml) were seeded into a 12-well culture plate and allowed to adhere for 2 h at 37°C. Non-adherent cells were carefully washed, and the adherent cells were stimulated for two days with isolated NETs (500 ng/ml), NETs with 40 IE/ml DNase1 (Dornase alfa, Pulmozyme®), dsDNA (250 ng/ml), or the vehicle control.

Liver-infiltrating neutrophil and macrophage isolation

Liver tissues were incubated in phosphate-buffered saline (PBS) containing 1 mg/ml Collagenase A (Roche) and 50 units/ml DNase I (Roche) for 30 min at 37°C. Digestion was quenched by adding FACS buffer (1 mM EDTA and 2% FBS in PBS), and single-cell suspensions were obtained by filtering through 70µm cell strainers (BD Biosciences). Erythrocytes were removed by a 5 min incubation in Pharm Lyse buffer (BD Biosciences). After washing with FACS buffer, neutrophils were isolated using a Human Neutrophil Enrichment Kit (Stemcell), while macrophages were isolated using a Human monocyte enrichment kit (Stemcell).

Ultrasound Examination

All US examinations were performed using an 18 MHz linear-array transducer (Aplio

i800, Canon Medical Systems). The rats were anesthetized with 1.5% isoflurane, positioned supine on a heated stage, and monitored via electrocardiogram. SWD imaging was performed with care to avoid the compression effect of the transducer. After the acquisition of the shear wave propagation data, the US system automatically displayed the dual view of grayscale images and shear wave propagation maps, and then switched to the quad-view mode including four maps (namely, the elasticity map, shear wave propagation map, grayscale image, and SWD map) after 5 seconds of cooling. The region of interest (ROI) was placed to align with the smooth and parallel lines on the propagation map, indicating stable measurement conditions. The SWD/SWE of livers and PVT were measured three times at each site from five independent images to increase reproducibility, with the average value being selected for subsequent analysis. Differentiation between the thrombus and the vessel wall was performed by the use of established parameters; the vessel wall is generally brighter (hyperechoic) than the incident thrombus. The thickest part of the venous wall was determined, and the portal vein wall thickness was measured from the posterior wall of the vein and recorded in millimeters. Intimal thickness measurements were taken between the inner hyperechoic line of the portal venous wall (tunica intima) and the inter end of the neighboring hypoechoic area (tunica media). Three measurements were taken for each, and the average value was recorded.

Endotoxin assay and Blood Biochemistry

Rat plasma was collected from whole blood by centrifugation at 3,000 rpm for 5 minutes. The concentrations of LPS in plasma and tissue homogenate were measured

by an endotoxin assay based on a limulus amebocyte extract with a chromogenic limulus amebocyte lysate (LAL) assay (Xiamen Huoshiji Cor. Ltd., Xiamen, China). All materials used for both sample preparation and testing were pyrogen-free. Samples were diluted in pyrogen-free water and heated to 70°C for 10 minutes to inactivate inhibitor agents that could interfere with the assay. All samples were tested in triplicate. The absorbance was measured by a microplate reader (Multiskan FC). The endotoxin content was expressed as endotoxin units per milliliter (EU/mL). Alanine aminotransferase (ALT) and aspartate aminotransferase (AST) levels were assessed using a commercially colorimetric assay (Elabscience Cat#E-BC-K235-M, and E-BC-K236-M, respectively).

Histology

Tissues were fixed in 10% formalin, paraffin-embedded, and 4 μm cross-sections were cut on slides. For staining, the section slides were deparaffinized (Safeclear II, Fisher Diagnostics, MI) and hydrated by graded ethanol washes. To evaluate the structure of PVT, sections from the proximal third of the thrombus were utilized. According to the manufacturer's protocol, sections were stained with hematoxylin and eosin (H&E, Solarbio, G1120), Sirius Red (SR, Solarbio, G1473), Masson trichrome (Masson, Solarbio, G1340), Martius Scarlet Blue (MSB, Solarbio, G2040), and elastic van Gieson (EVG, Solarbio, G1597). The collagen area was dyed blue by Masson, and ImageJ software was used to calculate the total thrombus cross-sectional area (μm2). The Threshold Color plugin was used to select collagen-stained areas and determine intrathrombus collagen content (μm2). EVG staining was used to identify

the elastic lamina within the vein, and ImageJ software was used to measure the intimal thickness. ImageJ software was used to measure the intimal thickness in each 400× image as follows: three measurements of intimal thickness were taken per image, for a total of 12 intimal measurements per section. Average intimal thickness was calculated for each thrombus-bearing rat.

Immunofluorescence

For immunohistochemical staining, sodium citrate buffer (pH=6) was used for heat-mediated antigen retrieval, and a solution containing 5% bovine serum albumin was used for blocking. The tissue sections were incubated overnight at 4°C with primary antibodies specific for ZO-1, ACTA2, CD68, COL1A1, H3-Cit, and MPO. For H3-Cit and MPO, secondary antibodies goat Anti-Rabbit Alexa Fluor 594 (Invitrogen) and goat Anti-Rat Alexa Fluor 488 (Invitrogen) were used for signal detection. Detection was performed using either biotinylated secondary Abs (goat anti-rabbit/rat, Abcam, 1:1000) or TSA Fluorescein (PerkinElmer, Waltham, Massachusetts, USA). For ACTA2, CD68, and COL1A1, Alexa Fluor™ 488 Tyramide SuperBoost™ kits Anti-Rabbit (Invitrogen, B40922), Alexa Fluor™ 594 Tyramide SuperBoost™ kits Anti-Rabbit (Invitrogen, B40925), and Alexa Fluor™ 647 Tyramide SuperBoost™ kits Anti-Rabbit (Invitrogen, B40926) were used. 4′,6-diamidino-2-phenylindole (DAPI) (Invitrogen, 62248) was used for counterstaining.

Protein isolation and immunoblotting

Total cellular proteins were isolated in RIPA buffer (ThermoFisher) with protease and phosphatase inhibitors (ThermoFisher), and 25-30 μg of the protein lysate was

separated on 4-20% TGX gel (Bio-Rad) and transferred to a 0.45 μm nitrocellulose membrane (Bio-Rad). The membranes were blocked in 5% BSA in TBST buffer and incubated with the following respective primary antibodies: ACTA2, H3Cit, GSDMD, Casps4, and Tubulin, overnight at 4°C. Goat anti-rabbit IgG and horse anti-mouse IgG were used as secondary antibodies, and blots were imaged using an enhanced chemiluminescence reagent (Amersham). Images were acquired on the PEQLAB Biotechnology system.

Scanning electron microscopy

Small pieces of collected thrombi were rinsed with saline and fixed with 2.5% glutaraldehyde for 30 minutes, dehydrated with 25%, 50%, 75%, and 100% ethanol for 10 minutes each time, critical-point dried, and then coated with 2 nm platinum (Pt-T4007, DM Material). After dehydration and critical-point drying, the specimens were analyzed under a scanning electron microscope (Helios G4 CX, ThermoFisher).

Quantitative reverse transcriptase polymerase chain reaction (qRT-PCR)

Total RNA was extracted by TRIZOL reagent. HiScript III All-in-one RT SuperMix (R333-01, Vazyme, Shanghai, China) was applied to synthesize complementary DNA (cDNA). mRNA levels were normalized to GAPDH. ChamQ SYBR qPCR Master Mix (Q341-02, Vazyme, Shanghai, China) was used for PCR amplification, and the relative gene expression level was calculated using a $2^{-\Delta\Delta CT}$ method.

Supplementary Table S1. Antibodies.

Name	Application and	Supplier	Cat No.

	dilution ratio		
ZO-1	IHC (1:500)	Abcam	ab221547
ACTA2	IF (1:50); WB (1:1000)	CST	19245
CD68	IF (1:500)	Abcam	ab283654
COL1A1	IF (1:500)	Abcam	ab270993
H3cit	IF (1:2000); WB (1:1000)	Abcam	ab281584
MPO	IF (1:200)	Abcam	ab300651
GSDMD	WB (1:1000)	Sigma Aldrich	G7422
Caspase-4	WB (1:500)	CST	4450S
β-tubulin	WB (1:1000)	Abmart	M20005
Anti-Mouse IgG; HRP-linked	WB (1:5000)	Abmart	M21001
Anti-Rabbit IgG; HRP-linked	WB (1:5000)	Abmart	M21002
goat anti-Rat Alexa Fluor 488	IF (1:200)	Invitrogen	A-11006
goat anti-Rabbit Alexa Fluor 594	IF (1:200)	Invitrogen	A-11012

Supplementary Table S2. Primers for QPCR.

Name	Sequence	Supplier

h-GAPDH-F	AACAGCCTCAAGATCATCAG	Sangon Biotech
h-GAPDH-R	AGTCCTTCCACGATACCAA	Sangon Biotech
h-TGF-β1-F	GGCCAGATCCTGTCCAAGC	Sangon Biotech
h-TGF-β1-R	GTGGGTTTCCACCATTAGCAC	Sangon Biotech
h-Col1a1-F	TGACCTCAAGATGTGCCACT	Sangon Biotech
h-Col1a1-R	ACCAGTCTCCATGTTGCAGA	Sangon Biotech
h-TNFα-F	TGGCGTGGAGCTGAGAGATA	Sangon Biotech
h-TNFα-R	TGATGGCAGAGAGGAGGTTG	Sangon Biotech
h-COL3A1-F	TGCCCACAGCCTTCTACACCT	Sangon Biotech
h-COL3A1-R	CAGCCATTCCTCCCACTCCAG	Sangon Biotech
h-SMAD3-F	CTACCAGTTGACCCGAATGTGC	Sangon Biotech
h-SMAD3-R	TCTGTCTCCTGTACTCCGCTCC	Sangon Biotech
r-GAPDH-F	TGCCACTCAGAAGACTGTGG	Sangon Biotech
r-GAPDH-R	TTCAGCTCTGGGATGACCTT	Sangon Biotech
r-TGF-β1-F	ATTCAAGTCAACTGTGGAGCAAC	Sangon Biotech
r-TGF-β1-R	CGAAAGCCCTGTATTCCGTCT	Sangon Biotech
r-IL1β-F	AAAAATGCCTCGTGCTGTCT	Sangon Biotech
r-IL1β-R	TCGTTGCTTGTCTCTCCTTG	Sangon Biotech

Supplementary Fig. S1: Representative H&E staining and Sirius red staining of a liver in the control and cirrhosis groups. Scale bar=200 μm.

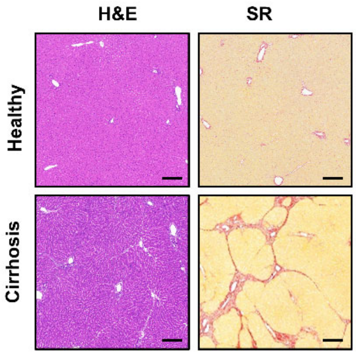

Supplemental Figure S2: Uncropped images of the western blot micrographs shown in Figure 3F.

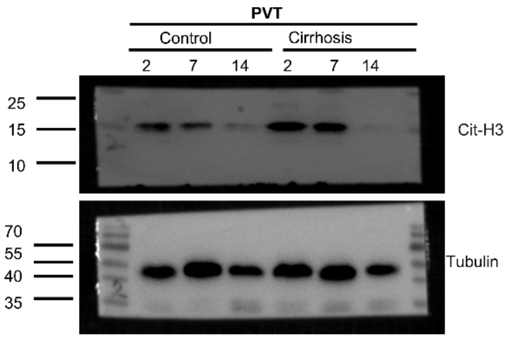

Supplemental Figure S3: Uncropped images of the western blot micrographs shown in Figure 3G.

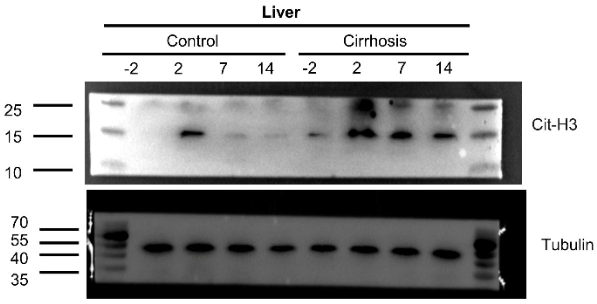

Supplemental Figure S4: Uncropped images of the western blot micrographs shown in Figure 4B.

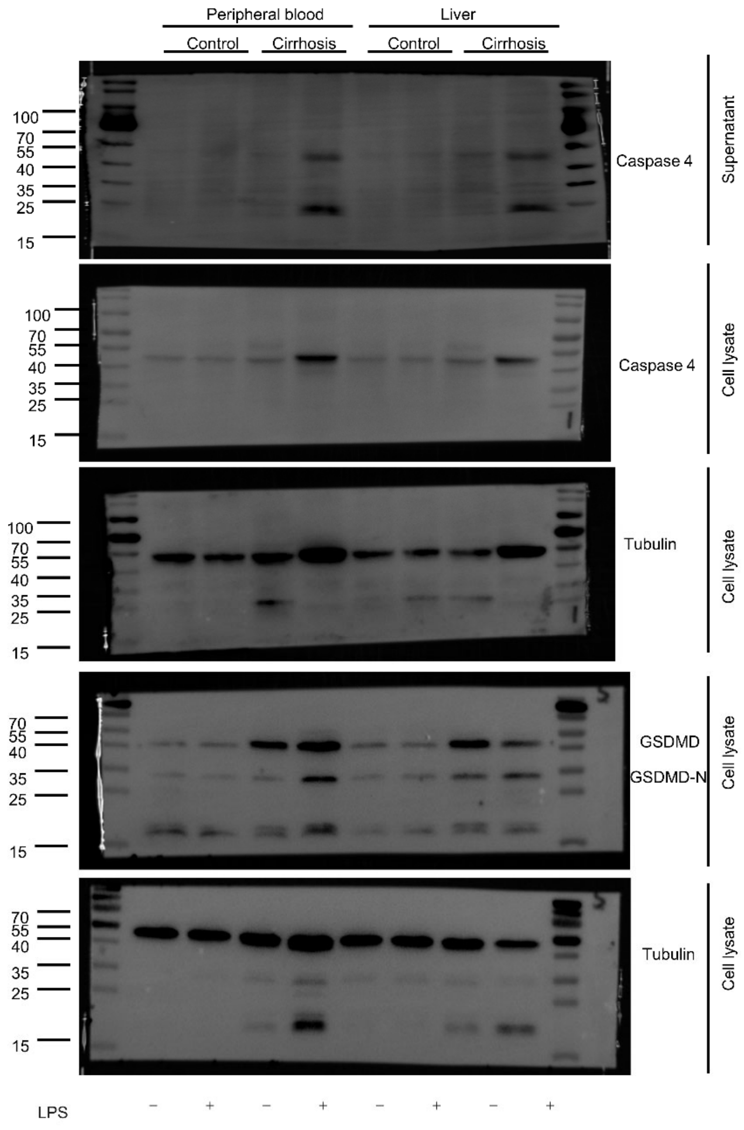

Supplemental Fig. S5: Uncropped images of the western blot micrographs shown in Fig. 4D.

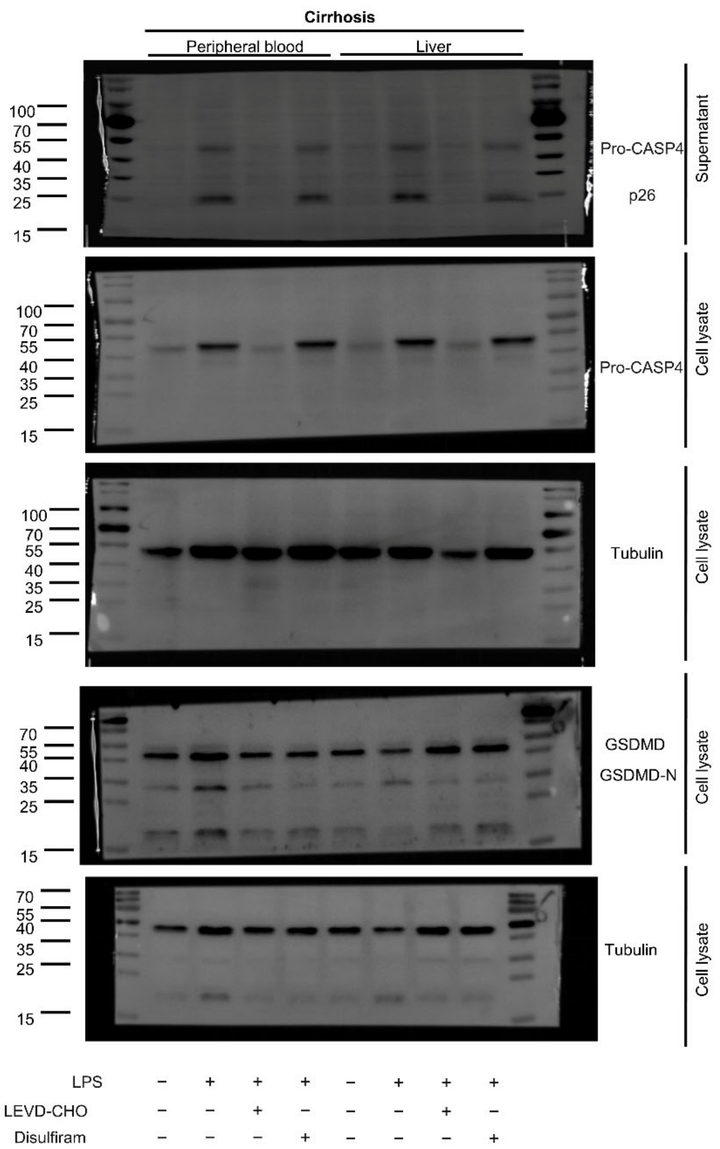

Supplemental Fig. S6: Uncropped images of the western blot micrographs shown in Fig. 5B.

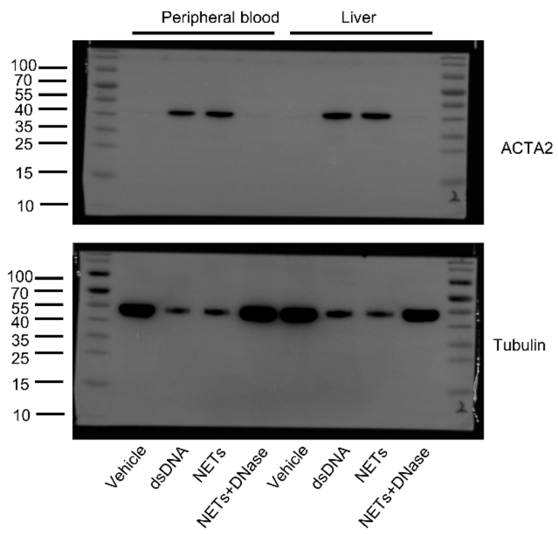

Supplemental Fig. S7: Uncropped images of the western blot micrographs shown in Fig. 6F.

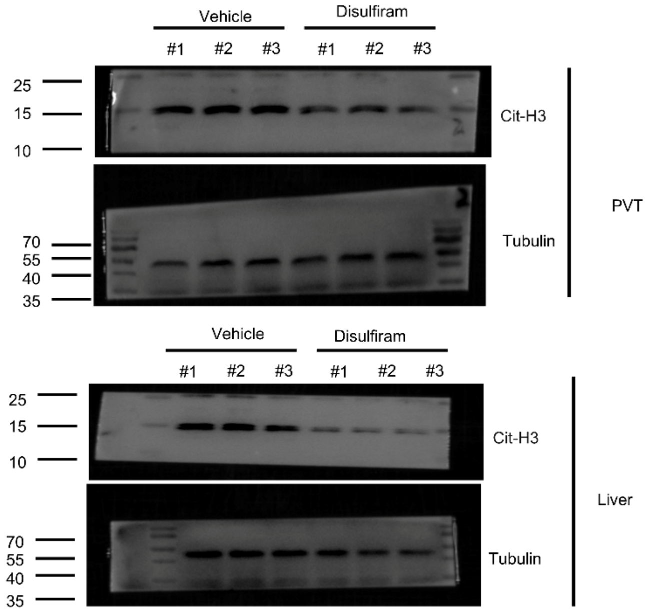

Supplement: Supplementary file 1 [file ijms-25-09099-s001.zip › ijms-3127071-supplementary.pdf]
